# Goal or Gold: Overlapping Reward Processes in Soccer Players upon Scoring and Winning Money

**DOI:** 10.1371/journal.pone.0122798

**Published:** 2015-04-15

**Authors:** Alexander Niklas Häusler, Benjamin Becker, Marcel Bartling, Bernd Weber

**Affiliations:** 1 Center for Economics and Neuroscience, University of Bonn, Bonn, Germany; 2 Department of Epileptology, University Hospital Bonn, Bonn, Germany; 3 Department of NeuroCognition/Imaging, Life&Brain Research Center, Bonn, Germany; 4 Department of Psychiatry, University of Bonn, Bonn, Germany; 5 Division of Medical Psychology, University of Bonn, Bonn, Germany; Inserm, FRANCE

## Abstract

Social rewards are important incentives for human behavior. This is especially true in team sports such as the most popular one worldwide: soccer. We investigated reward processing upon scoring a soccer goal in a standard two-versus-one situation and in comparison to winning in a monetary incentive task. The results show a strong overlap in brain activity between the two conditions in established reward regions of the mesolimbic dopaminergic system, including the ventral striatum and ventromedial pre-frontal cortex. The three main components of reward-associated learning i.e. reward probability (RP), reward reception (RR) and reward prediction errors (RPE) showed highly similar activation in both con-texts, with only the RR and RPE components displaying overlapping reward activity. Passing and shooting behavior did not correlate with individual egoism scores, but we observe a positive correlation be-tween egoism and activity in the left middle frontal gyrus upon scoring after a pass versus a direct shot. Our findings suggest that rewards in the context of soccer and monetary incentives are based on similar neural processes.

## Introduction

Seen as a driving force of human behavior and decision making, reward has previously been described as an operational concept for the positive value animals, including humans, ascribe to a behavioral act, object, or internal physical state [1]. While a lot of previous research has been dedicated to processing of primary rewards such as food, liquid, and sexual stimuli [2–4], secondary rewards of monetary and social nature are also very important motivators for human behavior [5–7]. Only two studies compared monetary and social reward processing in the form of positive social feedback and good reputation [8, 9]. In these studies, reward-related areas, i.e. the ventral striatum (VS) as well as the ventromedial prefrontal cortex (vmPFC), showed overlapping brain activity in response to monetary and social rewards [8, 9]. Results of these studies concurred with social exchange theory [10], which states that in social interactions not only materialistic goods such as money, but also non-materialistic goods such as social help are traded for other social goods as e.g. improved reputation.

Three major components are important in reward processing and reward-based learning: the probability, i.e. expectation of reward size and magnitude, the actual reward reception, and their difference: the reward prediction error [11, 12]. Previous studies comparing reward processing in a social and monetary context failed to investigate these different components in an active decision-making paradigm. We therefore developed a paradigm which allowed disentangling these different components in a social sport context by using reward in the form of goal-scoring in the most popular sport worldwide: soccer, which is played by about 265 million people around the globe (FIFA Communications Division (2007)—FIFA Big Count 2006: 270 million people active in football).

Our paradigm presents standard two-versus-one (2v1) situations in front of a goal in which subjects decide to either shoot to the goal directly or pass the ball to a team mate. By varying the situations and having them pre-rated by soccer experts, we manipulated the perceived probabilities of scoring the goal. The participant has a choice between a socially modest choice (pass) and a socially self-serving choice (shoot). The choice to pass to a teammate implies a more social, possibly team-oriented decision, while the decision to directly aim at the goal may add a personal on top of the social benefit. This is explained by pointing out that scoring a goal directly has an additional benefit to the goal scorer: while the team benefits from the goal, the scorer’s social reputation increases as well by directly becoming the focus point for celebration and social approval. This emphasizes that the decision to either pass or shoot is not solely influenced by the perceived probability of scoring the goal—but also by personality factors such as egoism, that determine whether social (prosocial) or personal (proself) benefits will be given greater weighting.

Two types of egoism have been previously identified: a hostile and derogatory kind, as well as a narcissistic and self-enhancement kind [13]. The Dutch Personality Questionnaire (DPQ) and Supernumerary Personality Inventory (SPI) egoism tests measure these two different kinds of egoism, which have been shown to converge on the Honesty-Humility (HH) scale of the HEXACO Personality Inventory—Revised (PI-R) [13]. The abbreviation HEXACO stands for the six dimensions of the PI-R: “Honesty Humility”, “Emotionality”, “eXtraversion”, “Agreeableness”, “Conscientiousness”, and “Openness to Experience”, with the test being freely accessible online (hexaco.org). It is based on the Five Factor Model and it is the HH scale which has been established as a sixth dimension which lies beyond “the Big Five” [13]. Specifically, the HH assesses egoism on a behavioral dimension ranging from sincere, modest, and fair (when scoring low) to insincere, greedy, and boastful (when scoring high) [13–17]. It might therefore be ideally suited to assess the personality trait egoism in the context of the present research question. Another well-established measure to explain social behavior by distinguishing between prosocial and proself orientations is the social value orientation (SVO) questionnaire [18]. Using this questionnaire, previous studies were able to identify a correlation between it and the HH scale of the HEXACO PI-R [15].

By means of an active decision making soccer paradigm and a well-established monetary incentive task, we examined overlapping brain activation during monetary and soccer-specific social reward processing, including reward probability, reward reception and reward prediction errors. Additionally, we hypothesize egoism to correlate with stronger reward related signals upon scoring a goal after a direct shot versus after a pass to a teammate. On the behavioral level we expect to find more egoistic players to shoot the ball significantly more than to pass it.

## Materials and Methods

### Pre-testing

**Fig 1:**
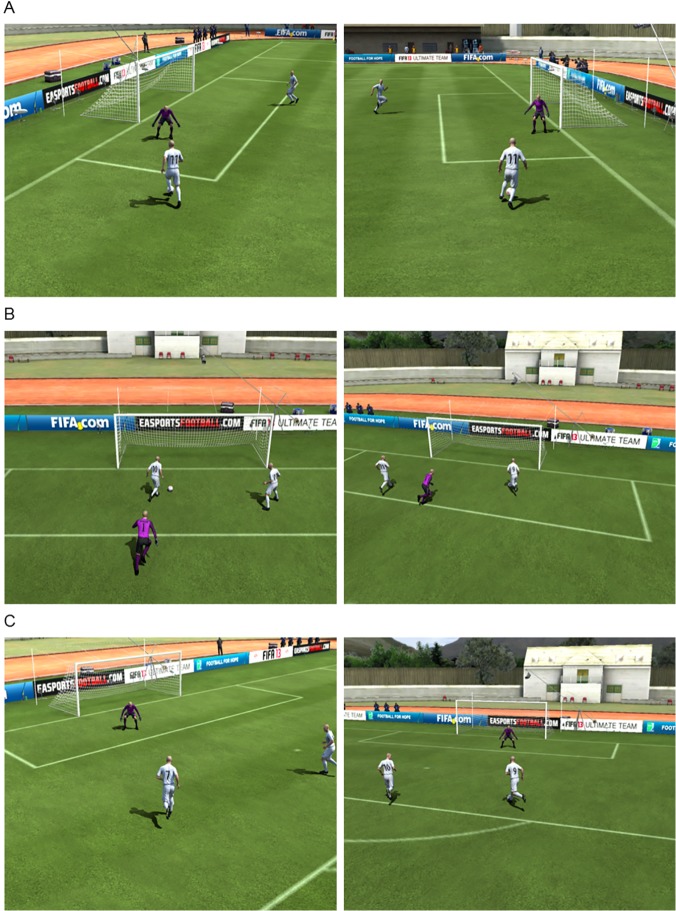
Soccer situations. Six exemplary images of the different soccer situations presented in the soccer paradigm. The player in ball possession approaches the goal from either the left or the right side of the penalty spot. A. Clear situation (pass). B. Clear situation (shoot). C. Unclear situation.

Pre-testing stimuli for the soccer paradigm were created by taking screenshots (1400x1050 pixels, 4:3 format, window mode, rendering quality: high, MSAA option: off, frame rate: no limit) of 200 different 2v1 situations from the soccer simulation FIFA 13 (Electronic Arts Inc., Redwood City, CA, USA). Screenshots displayed standardized 2v1 situations involving two attacking players with ball possession and a defending goalkeeper from the opposing team (Fig 1 and S1 Supporting Information). Pre-testing was performed using the online survey tool Qualtrics (Provo, UT, USA). For each image, participants were asked three randomly presented questions:

1. How likely is it that you shoot the ball yourself, rather than passing it to your teammate, in order to score?
2. How likely is it for you to score upon shooting the ball?
3. How likely is it for your teammate to score after having received a pass?

377 German soccer players rated the situations. These soccer players were recruited by distributing the Qualtrics online link via Email to all of the 262 German regional soccer associations, while the Berlin and Hamburg soccer clubs were written to individually. Only teams and officials from the Middle Rhine soccer association were not contacted to keep this pool of participants for the fMRI part of the experiment. Furthermore, the link was posted online in local soccer blogs and social platforms. Each situation was rated an average of 38.63 (± 3.44) times. Question one ratings were used to categorize the situations into the 40 most unclear and 20 clearest situations (10 for shot and pass, respectively) and question two and three ratings were used to determine the scoring probabilities.

### Soccer Paradigm

**Fig 2:**
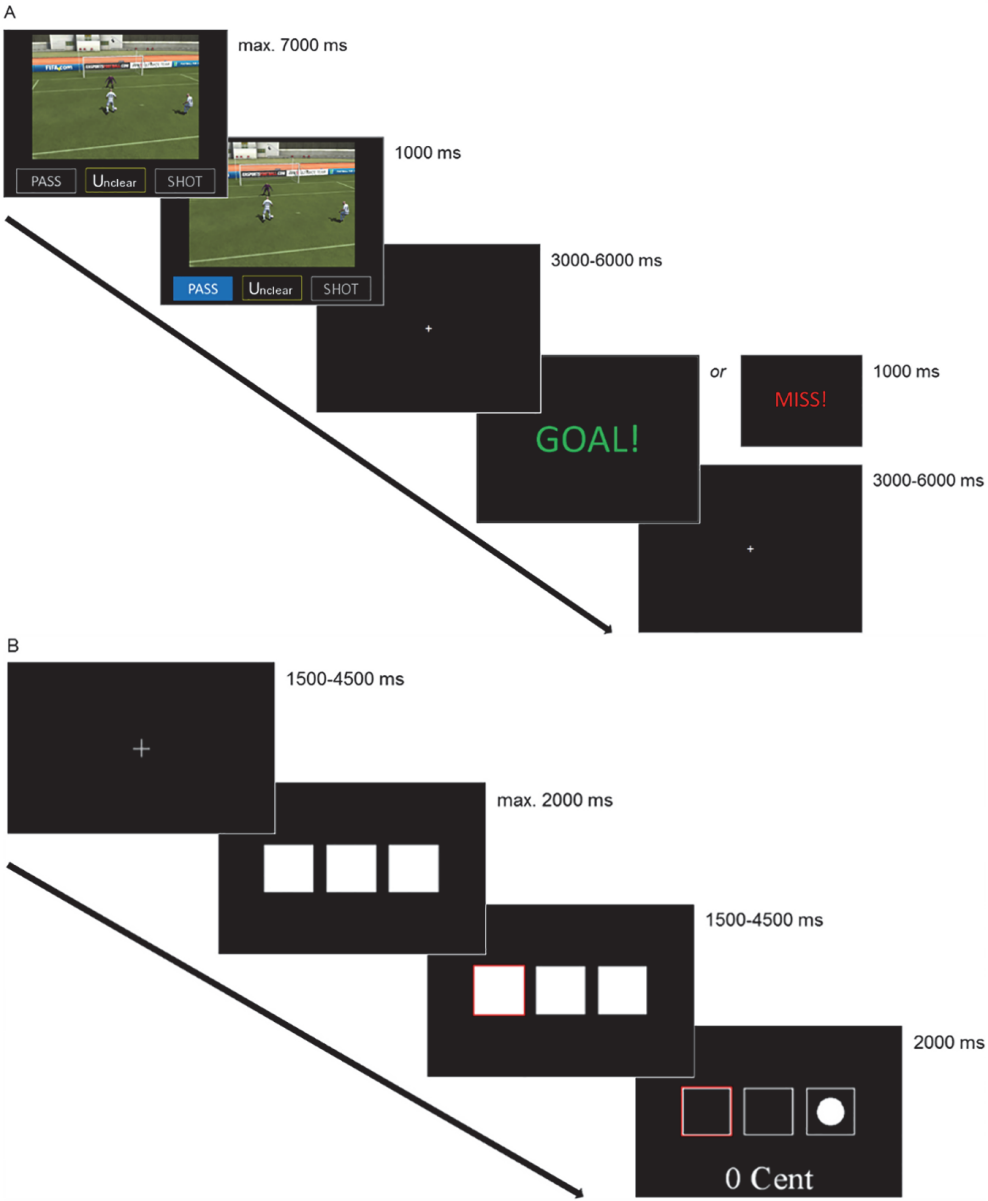
The soccer and monetary paradigm. Timeline for an exemplary trial out of the 120 trials shown in the soccer paradigm and the 150 trials shown in the monetary paradigm.

The fMRI paradigm was programmed using in-house software. Upon being confronted with the situation, participants decided to either pass or shoot the ball via button press (Fig 2A). Each situation was randomly shown twice and half of the feedbacks were preset to be positive (GOAL!) and the other half negative (MISS!), thus leading to 60 goals and 60 misses, respectively. The stimuli had an interstimulus interval (ISI) and intertrial interval (ITI) of 3000–6000 ms programmed for randomization using the “randint” function of Python 2.7 (Python Software Foundation, Beaverton, OR, USA). The feedbacks were preset in reference to the ratings of pre-testing questions two and three, thus making the feedback as realistic as possible.

### Monetary Paradigm

In the previously published monetary incentive paradigm [19, 20], participants guessed under which out of one to four randomly shown boxes a circle was hidden, leading to winning probabilities ranging from 25% to 100% (Fig 2B). In each of the 150 trials, a correct guess led to a positive monetary feedback (win) of 10 euro cents and a wrong guess to no monetary win (no win), while no guess led to a monetary loss of 10 euro cents. The ISI and ITI were also programmed for a randomization of 1500–4500 ms using Python 2.7.

### Participants

33 male (age: 24.39 ± 3.20 years) participants were recruited from local soccer teams via internet advertisements, flyers, word of mouth, and personal recruitment sessions at local soccer clubs. Exclusion criteria were a history of neurological or psychiatric disorders, involvement in the online pre-testing questionnaire, as well as conditions prohibiting the participation in an MRI setting. Additionally, participants had to be right-footed and actively playing at a soccer club. The participants received a show-up fee of 10 euro as well as additional monetary compensation depending on the results of the monetary paradigm and the monetary incentivized SVO test. All participants gave written informed consent according to the Declaration of Helsinki (BMJ 1991; 302; 1194) and the experiment was approved by the ethics committee of the University of Bonn.

### Experimental Procedure

#### Personality Questionnaires

Each participant handed in the completed personality questionnaires (HEXACO PI-R 200 and SVO, previously distributed) at the scanning appointment. Additionally, a questionnaire regarding personal data and soccer experience was filled out by the participants on site (S1 Table). They subsequently received detailed instructions about the experimental procedure as well as ethical and medical implications. As part of an oral briefing procedure taking place directly ahead of entering the scanning room, each subject was instructed to not view the scenes as being from a video game, but rather as real-life situations with the teammate and opposing goalkeeper possessing real-life flaws.

#### fMRI Experiment

In total the experiment took two hours, consisting of three parts: a psychological questionnaire (~30 minutes), scanning preparation (~30 minutes), and scanning session (max. 90 minutes). The scanning session consisted of the soccer paradigm (max. 34 minutes), monetary paradigm (max. 32 minutes) and the final structural T1 measurement (~9 minutes). Participants were scanned on a 1.5 T Avanto Scanner (Siemens, Erlangen, Germany). Instructions were given regarding the emergency ball, the use of OHROPAX Classic ear protection (OHROPAX GmbH, Wehrheim, Germany), and the correct button pressing via the respective response grips (Nordic NeuroLab, Bergen, Norway). An 8-channel head coil was placed above the participant’s face and video goggles (Nordic NeuroLab, Bergen, Norway) used to present the stimuli were installed on the head coil. Each paradigm was presented using Presentation v14.9 (NeuroBehavioural Systems Inc., Albany, CA, USA). Following the scanning sessions, participants were debriefed with regard to the preset soccer feedbacks and the study objective concerning egoism.

### Imaging Protocol

Acquisition of the functional data was done using EPI-sequences with a repetition time (TR) of 2.5 s, echo time (TE) of 45 ms, and a flip angle of 90 degrees. The image resolution was 64 x 64 pixels and the field of view 192 x 192 mm. 31 slices covering the brain from the superior part of the cerebellum to the top of the cerebrum including the midbrain were obtained in an axial fashion and an interslice gap of 0.3 mm. This resulted in a voxel size of 3 x 3 x 3.3 mm.

### fMRI Analyses

Data sets of five participants were excluded due to one participant not fully understanding the monetary paradigm, one scanning session being cancelled because of technical problems, one participant having a metal plate inside his knee, and data sets of two participants showing excess head motion (translational: >3 mm, rotational: >2.5 degrees). Statistical Parametric Mapping 8 (SPM8, Wellcome Department of Imaging Neuroscience, London, UK) was used to analyze the data sets of the remaining 28 participants (age: 24.57 ± 3.21 years).

Pre-processing steps included slice time correction, motion correction, spatial normalization to the T1 image of each participant, reslicing to a 3 x 3 x 3 mm voxel size, and a final smoothing step using a Gaussian kernel with full-width at half-maximum (FWHM) of 8 mm. Creating an identical GLM for both paradigms was technically not possible due to the necessity of combining the positive and negative feedback as well as the combined parametrically modulated (via the reward prediction error (RPE)) feedback in one regressor each. Therefore, brain activation was estimated using a total of four general linear models (GLM): two soccer and two monetary GLMs with GLM-specific regressor combinations.

GLM1 (soccer paradigm):

- 1. Choice
- 2. Choice (parametrically modulated via reward probability (RP))
- 3. Positive feedback (goal) after a pass
- 4. Positive feedback (goal) after a shot
- 5. Negative feedback (miss)
- 6. Missed response
- 7.-12.: Movement regressors

GLM2 (monetary paradigm):

- 1. Choice
- 2. Choice (parametrically modulated via RP)
- 3. Positive feedback (win)
- 4. Negative feedback (no win)
- 5. Missed response
- 6.-11.: Movement regressors

GLM3 (soccer paradigm) and GLM4 (monetary paradigm):

- 1. Choice
- 2. Feedback
- 3. Feedback (parametrically modulated via reward prediction error (RPE))
- 4. Missed response
- 5.-10.: Movement regressors

The RP parameter in the soccer paradigm was calculated based on the scoring probabilities assessed in the pretest; the reward probabilities in the monetary paradigm were directly related to the number of boxes shown in each trial (i.e. 0.25, 0.33, 0.5 or 1). The RPE parameter was computed as the difference between the outcome (i.e. 1 or 0) and the given RP. All of the regressors were convolved with the canonical hemodynamic response function (HRF) as implemented in SPM8. First-level contrasts were then established for each of the four GLMs and used for the second-level random effects analyses (p<0.001, uncorrected). These included one sample t-tests for the ‘‘RP versus zero’ (one for each paradigm), ‘goal versus miss’/’win versus no win’ (reward reception), ‘goal after a shot versus goal after a pass’, ‘goal after a pass versus goal after a shot’, as well as ‘RPE versus zero’ (one for each paradigm) contrasts. A whole brain search was performed for each contrast and activities were listed accordingly (S4 and S5 Tables). The coordinates and T values of the peak voxels were determined in SPM8 and the relevant regions were then resolved using the automatic anatomic labeling (aal) atlas [21], as implemented in the xjView toolbox (available via http://www.alivelearn.net/xjview). By using this atlas, brain regions were inspected individually and labeled according to the terms used in the literature. The exact procedural description and an abbreviation overview can be found in the supplementary information (S8 Table). All of the beta and contrast images for each of the 28 subjects are freely accessible via the Harvard Dataverse Network website at “http://thedata.harvard.edu/dvn/dv/ANH”.

The differential activation was statistically compared using the second-level specification feature of SPM8. For this purpose, each of the three specific reward contrast images (RP versus 0, RR, and RPE versus 0) were compared via paired t-tests. After subsequent estimation, the contrasts of soccer versus monetary reward activity were compared on a whole-brain level (k >10, df = 27, S6 Table).

The egoism analysis was performed by using the egoism values from the Honesty-Humility scale of the HEXACO 200 PI-R as covariates in the contrasts “scoring after a shot versus a pass” and “scoring after a pass versus scoring after a shot”. Additionally, behavioral egoism values were calculated by taking the proportion of shots in the clear situations normalized by the number of total shots and integrating them as covariates in the same contrasts described above. All behavioral data was analyzed using IBM SPSS Statistics 21 (IBM Corp., Armonk, NY, USA).

### ROI Analyses

**Fig 3:**
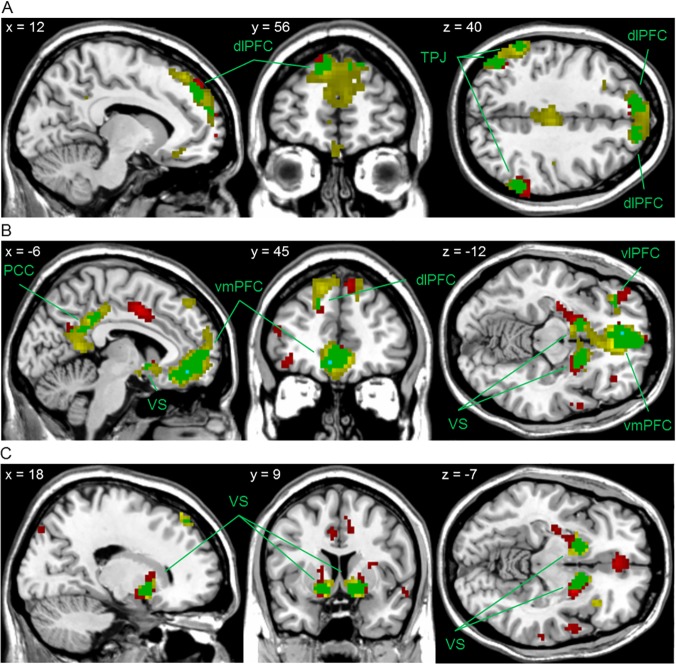
Masks of the reward processing regions activated in each and both paradigms (k > 10, df = 27). A. Reward Probability (RP) versus 0. B. Reward Reception (RR): Win/Goal > NoWin/Miss. C. Reward Prediction Error (RPE) versus 0. Yellow: monetary, p<0.001, uncorrected. Red: soccer, p<0.001, uncorrected. Green: areas activated in both paradigms, p<0.001, uncorrected. Turquoise: areas activated in both paradigms, p<0.05, FWE-corrected. Abbreviations: dlPFC: dorsolateral prefrontal cortex, PCC: posterior cingulate cortex, TPJ: temporal parietal junction, vlPFC: ventrolateral prefrontal cortex, vmPFC: ventromedial prefrontal cortex, VS: ventral striatum.

In line with a previous study investigating overlapping brain activation [8], regions of interest (ROI) masks were created from the monetary reward contrasts ‘RP versus 0’, ‘win versus no win’, and ‘RPE versus 0’ using the xjView toolbox (p<0,001, k>10, Fig 3 (yellow color)). More details of the regions included in the monetary ROI masks and the results of the whole brain search for each of paradigms can be found in the supporting information (S4 and S5 Tables, as well as S2 Supporting Information). The monetary masks were then applied to the respective soccer brain activation contrasts (p<0.001, k>10, relevant masks can be seen in Fig 3 (red color) and S2 Supporting Information).The results were small volume corrected (p<0.05, familywise error (FWE)-corrected) and checked for significantly overlapping active regions (Table 1). Additionally, four ROI masks were obtained from the authors of the previously published paper involving the monetary paradigm [20]. These were based on the Oxford-Harvard cortical and subcortical atlases and included the bilateral ventral striatum, the ventral midbrain, and the medial orbitofrontal cortex. These ROI masks were however only used to check for the independent activation robustness of the monetary paradigm and are considered irrelevant for the main part of the analysis.

**Table 1:** Overlapping activity in both tasks: Small volume corrected brain activation upon soccer and monetary reward probability (RP), reward reception (RR), and reward prediction error (RPE) (k >10, df = 27).

**Contrast**	**Region**	**Laterality**	**MNI coordinates**			**Cluster size**	**T**
			x	y	z		
****Reward probability****	TPJ***	L	-51	-70	34	192	6.26
	dlPFC*	L	-15	50	37	65	5.53
	TPJ*	R	60	-52	43	37	5.36
	POG*	R	45	-25	64	55	5.01
	TPJ**	L	-60	-49	43	48	4.70
	dlPFC*	R	18	50	40	25	4.54
****Reward reception****	vmPFC***	L	-3	47	-8	310	6.57
	VS**	R	24	5	-11	61	6.13
	VS**	L	-12	11	-5	42	5.69
	PCC***	L/R	0	-46	28	189	5.54
	dlPFC**	L	-18	32	52	52	5.09
	vlPFC*	L	-36	38	-8	20	4.73
****Reward prediction error****	VS***	R	21	2	-11	58	7.20
	VS**	L	-18	2	-11	45	5.33

ROI masks were created from the monetary contrasts ‘RA versus 0’, ‘win versus no win’, and ‘RPE versus 0’, respectively. The activity during soccer reward processing was then corrected for by small volume using the respective masks. Abbreviations: dlPFC (dorsolateral prefrontal cortex), PCC (posterior cingulate cortex), POG (postcentral gyrus), TPJ (temporal parietal junction), vlPFC (ventrolateral prefrontal cortex), vmPFC (ventromedial prefrontal cortex), VS (ventral striatum).

*** p(FWE-corr.)<0.001,

** p(FWE-corr.)<0.01,

* p(FWE-corr.)<0.05.

## Results

### Participants and Personality Test Results

The most competitive player was active in the 5th German league (Mittelrheinliga), while most participants (n = 10) played in the 9th German league (Kreisliga B; S1 Table). The HEXACO PI-R 200 was analyzed on each domain and facet-level scale (S2 Table), resulting in only four participants being categorized as egoistic. Using the data from the SVO questionnaire, 21 prosocials, five proselfs, and no competitors were identified (mean = 1.19, SD = 0.40), while two were not classified due to inconsistent responses. Only one participant was characterized as egoistic and proself. Subsequent analysis using the SVO scores was refrained from due to lack of statistical power.

### Behavioral Results

Behavioral data from the soccer paradigm was grouped into four categories. For each of the clear and unclear categories, pass and shoot choices were analyzed (S3 Table). Out of the 3360 situations, only five situations were not responded to. Overall, soccer players did not decide to shoot significantly more or less than to pass the ball (S3 Table). In order to identify individual soccer players that chose to either shoot or pass more than others, one SD above/below the average ratio was used as a threshold. This resulted in five participants having chosen to shoot directly significantly more than the rest.

### fMRI Results

#### Reward Probability

Overlap analysis using inclusive masking revealed overlapping brain activity modulated by reward probability in the bilateral temporal parietal junction (TPJ), bilateral dorsolateral prefrontal cortex (dlPFC), and right postcentral gyrus (POG) (Table 1 and Fig 3A, green color). No differential activation was observed.

#### Reward Reception

The very medial posterior cingulate cortex (PCC), bilateral ventral striatum (VS), left dlPFC, left ventrolateral prefrontal cortex (vlPFC) and left vmPFC were all found to be significantly active in both types of reward reception (Table 1 and Fig 3B, green color). The vmPFC activation was found to extend into the subgenual and rostral anterior cingulate cortex (sgACC and rACC, respectively), as well as into the medial orbitofrontal cortex (mOFC). Only scoring a goal versus missing led to additional significant brain activity (S6 Table). This was found in the bilateral TPJ, as well as in areas of the left vlPFC.

#### Reward Prediction Error

Overlapping activation during reward prediction error processing was observed in the bilateral VS (Table 1 and Fig 3C, green color) and no differential activation was observed.

#### Correlation to Egoism

**Fig 4:**
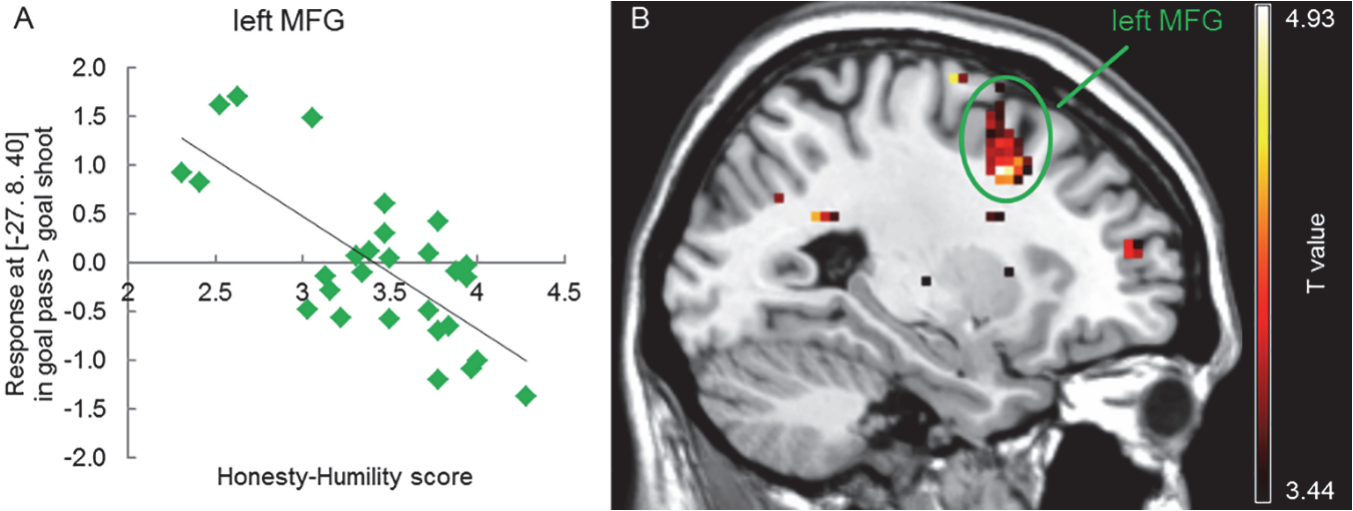
Positive correlational activity between egoism and scoring after a pass versus a shot at the left middle frontal gyrus (MFG, k >10, df = 27). The negative correlation of brain activity and Honesty-Humility depicts a positive correlation with egoism. A. Correlational analysis of Honesty-Humility scores and contrast values at the left MFG cluster. B. left MFG (green circle) activation in correlation to egoism upon scoring after a pass versus a shot.

Contrasting scoring a goal after a shot versus a pass and vice versa led to no significant activation. Furthermore, the behavioral egoism covariate analyses did not show significant brain activation. No correlating brain activation was observed between egoism scores (Honesty-Humility scale of the HEXACO PI-R 200) and brain activation due to “scoring after a shot versus scoring after a pass”. However, a negative correlation was found between the Honesty-Humility scale of the HEXACO PI-R 200 and brain activation (i.e. positive correlation of egoism) upon “scoring after a pass versus scoring after a shot” (S7 Table and Fig 4A) in the left middle frontal gyrus (MFG; p(FWE-corr.) = 0.002; S7 Table and Fig 4B).

## Discussion

Using the combination of a standard monetary incentive and a newly developed soccer-related task, we were able to investigate different computational aspects in reward-based learning in a soccer-specific social in comparison to a monetary context, i.e. reward probability, reward reception, and reward prediction errors. Our data does not only show a strong overlap of the neural substrates involved in monetary and soccer-specific social feedback in different aspects of reward processing, but also differential activation during reward reception.

### Reward Probability

The bilateral dorsolateral prefrontal cortex (dlPFC), temporal parietal junction (TPJ), and right postcentral gyrus (POG) were shown to be active during anticipation of both reward types. The dlPFC has been associated with social judgment and decision making, specifically in relation to behavioral control of social strategic behavior [22], implementation of fairness-related behavior [23], and social norm compliance [24, 25]. The TPJ has been shown to be involved in spatial working memory tasks [26] as well as social contexts such as apology and forgiveness [27], as well as attention and social cognition [28, 29]. Additionally, right POG activation was observed during the reward anticipation in both paradigms, a region that has identified as the primary somatosensory cortex [30].

### Reward Reception

The ventromedial prefrontal cortex (vmPFC), bilateral ventral striatum (VS), posterior cingulate cortex (PCC), as well as the left dorsolateral prefrontal cortex (dlPFC) and left ventrolateral prefrontal cortex (vlPFC) were involved in reward reception in both paradigms. The largest cluster peaking in the vmPFC and extending into the subgenual and rostral anterior cingulate cortex (sgACC and rACC, respectively), and medial orbitofrontal cortex (mOFC), as well as the activation of the VS have been associated with social and monetary reward processing in other previously mentioned studies comparing social and monetary reward [8, 9]. Besides the ventral parts of the striatum having long been known to be part of the reward circuit [31–33], the sgACC and rACC could have played a role in the attention circuit regulating cognitive and emotional processing [34, 35]. This is also relevant in pointing out that similar activation in these two regions was observed in another soccer reward processing study that contrasted goal and miss, as well as goal versus open play [36]. To our knowledge this is the first study showing evidence for a direct overlap in the vmPFC and VS with respect to monetary and soccer-specific social rewards, even though other studies have shown involvement in social reward processing as well [5, 8, 9, 37, 38]. The PCC has been previously shown to be linked to value association in connection with reward processing [39], as well as episodic memory retrieval [40], and visuospatial attention [41, 42]. It is therefore difficult to interpret the exact role in our paradigm. Specifically, activations of the left dlPFC have been linked to behavioral control of strategic social behavior [22] and attention control during task preparation [43], while left vlPFC activation has been related to cognitive control of current relevant memory [44], and selection between competing active representations as used in goal-selection [45, 46].

The direct comparison of reward-related activity in both tasks revealed more activation in the soccer as compared to the monetary paradigm. Of these, the TPJ has been associated with spatial working memory tasks [26], suggesting a stronger involvement possibly occurring due to expertise of the soccer players with these standard situations and therefore implicit spatial memory retrieval. Additionally, the TPJ has been associated with social contexts, such as social cognition in relevance to attention [28], apology and forgiveness [27], and parochial punishment [29]. The differential activation of the TPJ only existing in relevance to the soccer paradigm could therefore be suggested to represent the sport’s implicit social context as shown in the form of 2v1 situations. Additionally, activation only found upon scoring a goal was also located in the left vlPFC. As mentioned before, this region is especially activated during cognitive control of current relevant memory [44] and selection between competing active representations as used in goal selection [45, 46]. It can henceforth be suggested that processing these features of the soccer situations require greater neural effort than simpler guessing situations such as shown in the monetary paradigm. It is important to note however that such a suggestion can only be finalized following a same study procedure involving “non-experts”.

### Reward Prediction Error

The bilateral VS was significantly activated during reward prediction error processing in the soccer game as well as in the monetary domain. It is well known for its role in reward prediction error processing [19, 20, 37, 47–49], but this is the first time a sport-related context has been shown to elicit this type of activity.

### Egoism

The whole HEXACO test was handed out to the participants to prevent the soccer players from recognizing the egoism component of the study and thus influencing the decision making process of the individuals. This was especially important since egoism is known to be associated with negative personal features such as antisocial behavior [13]. Even though no correlation between egoism and brain activation upon scoring a goal after an own shot and scoring after a pass was observed, we did find a positive correlation between egoism and activation in the middle frontal gyrus (MFG) in response to scoring a goal after a pass versus after a shot. This region has been linked to many different higher cognitive processes such as image recognition [50], spatial working memory tasks [26], emotional processing of happiness [51], reasoning [52], as well as future event construction and elaboration [53], but has not been shown to be involved in reward processing, specifically. This rather counterintuitive finding might be explained by suggesting that more egoistic soccer players do not require any neural effort to process scoring a goal after a shot since these individuals value this as a rather normal situation, while scoring after passing the ball to a teammate requires self-reflective spatial and reasoning neural effort in regions such as the MFG. By not finding significant activation in the previously mentioned reward areas, our data does not support the hypothesis of egoism correlating with stronger reward related signals upon scoring a goal after a direct shot versus after a pass to a teammate. Additionally, our behavioral observations do not show a correlation between passing and shooting behavior in 2v1 situations in front of goal and egoism. These results can be seen as a first step towards dealing with individuals in team sports who thus far have been labeled by the public and media as ‘egoistic’. Even though we did not find any correlation between egoism scores and passing versus shooting behavior, it is too far-fetched to suggest the personality trait egoism to not guide the oftentimes labeled selfish decisions on a soccer pitch. Future studies involving other in-game soccer situations (e.g. in the middle of the pitch decisions: ‘dribble versus pass’) and other team sport decision making paradigms should therefore be done in order to determine the personality and social aspects guiding such behavior. Determining these aspects could be crucial in developing guidelines for coaches and teammates to deal with mistakenly labeled ‘egoistic’ individuals in social activities such as team sports.

### Limitations

Even though we think that a soccer-related task such as the one implemented in the experiment has an inherent social context, it is important to point out that there are several possibilities for improving the social aspect of the experimental setup. Since the participant in the scanner does not interact with other participants directly, creating an avatar of the participant and one of his teammates could serve as an idea for a future study. With the participant then lying in the scanner, the teammate could observe the decisions made by the participant from the scanner operating room and respond accordingly, thus giving social feedback, or two players could directly interact in a hyperscanning experiment. As recently published in relation to social feedback processing, another idea would be to implement a camera in the scanner in order to emphasize the social aspect of the soccer-specific social experimental procedure even further [54].

## Conclusion

Our results suggest strong overlapping neural processes underlying reward probability, reward reception, and reward prediction error processing during highly motivating team sport situations and monetary incentives. While reward reception and reward prediction error processes overlap in reward related regions, reward probability processing requires higher cognitive effort in both domains. Besides extending this research to other reward-related sport activities and to different levels of sport expertise, future research could investigate the differences and similarities of reward paradigms in order to decipher the exact processing components of decision making during reward anticipation and probability. Our results furthermore suggest that the role of the term egoism in team sports should be further researched on in order to possibly support the finding of egoism failing to be shown as a driving force of on-pitch soccer behavior.

## Supporting Information

S1 Supporting Information Screenshot specifications. (DOCX)

S2 Supporting Information Detailed region of interest specifications relevant to the mask created via the monetary paradigm (p<0.001, k>10, uncorrected). (DOCX)

S1 Table Physical, soccer, and video game attributes of 28 soccer players. (DOCX)

S2 Table HEXACO PI-R 200 domain and facet-level scale values of 28 soccer players. (DOCX)

S3 Table Behavioral data from the soccer paradigm, displaying number of shots and passes in the given 80 unclear and 40 clear situations. (DOCX)

S4 Table Brain activity related to soccer reward probability, reward reception, and reward prediction error (k >10. df = 27). (DOCX)

S5 Table Brain activity related to monetary reward probability, reward reception, and reward prediction error (k >10, df = 27). (DOCX)

S6 Table Differential Brain activity upon contrasting soccer versus monetary reward reception (paired t-test, k >10, df = 27). (DOCX)

S7 Table Negative correlation between the HEXACO PI-R 200 Honesty-Humility scale and brain activity at scoring after a pass versus scoring after a shot (k >10, df = 27). (DOCX)

S8 Table Brain region name abbreviations. (DOCX)
