# Supplementary material for: Goal or Gold: Overlapping Reward Processes in Soccer Players upon Scoring and Winning Money
Source: PLoS One. 2015 Apr 15;10(4):e0122798. doi: 10.1371/journal.pone.0122798 (PMC4398371; doi:10.1371/journal.pone.0122798)
Supplement: S1 Supporting Information — (DOCX) [file pone.0122798.s001.docx]

**Supporting Information S1.** Screenshot specifications.

The following details were used to create the screenshots for the two-versus-one situations:

- Attacking players:
  - White short-sleeve jerseys, white shorts, white socks, and black cleats,
  - Black numbers 7, 8, 9, 10 or 11 on both jerseys and shorts.
- Goalkeeper:
  - Purple/black long-sleeve jersey,
  - Black number 1, black/purple shorts, black/purple socks, and black cleats.
- No brand names or logos were displayed in any form.
- Point of view: always from behind the player in possession.
- No offside situations.
- No fans were included in the images.
- Background scenery involved advertising boards displaying only “FIFA 13 ULTIMATE TEAM”, “FOOTBALL FOR HOPE”, “FIFA.COM”, and “EASPORTSFOOTBALL.COM”.
- Further components of the images were:
  - Running track,
  - Photographers,
  - Cameras,
  - Chairs,
  - Forest,
  - Fence,
  - White house.
