# Supplementary material for: Goal or Gold: Overlapping Reward Processes in Soccer Players upon Scoring and Winning Money
Source: PLoS One. 2015 Apr 15;10(4):e0122798. doi: 10.1371/journal.pone.0122798 (PMC4398371; doi:10.1371/journal.pone.0122798)
Supplement: S2 Supporting Information — (DOCX) [file pone.0122798.s002.docx]

**Supporting Information S2.** Detailed region of interest specifications relevant to the mask created via the monetary paradigm (p<0.001, k>10, uncorrected).

Figure SI2 and Table SI2 relate to each other, with Figure SI2 displaying the activation listed in Table SI2 using the glass-brain view implemented in xjView.

**Figure SI2. Brain activation during the three different phases of monetary reward processing (p<0.001, k > 10, df = 27).** The red arrow indicates the respective locations of the global maximum.

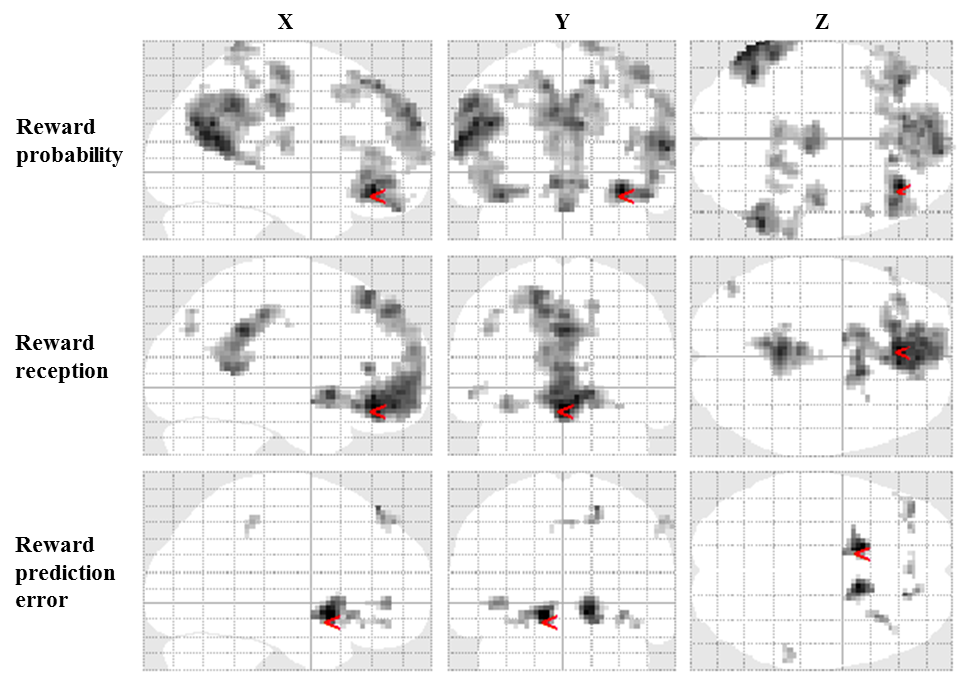


**Table SI2.** Brain activity related to monetary reward anticipation, reward reception, and reward prediction error (p<0.001, k >10, df = 27). All regions were defined using the automatic anatomic labeling (aal) toolbox [[28](#_ENREF_28)] and activity in the ventral anterior putamen and caudate nucleus was summarized as ventral striatal activity. The top three clusters of each peak location are displayed and sorted according to cluster size.

| **Contrast** | **Cluster number** | **Total number of voxels** | **Maximum peak intensity (T)** | **Peak MNI coordinates** | | | **Region** | **Late-**  **rality** | **Cluster size** |
| --- | --- | --- | --- | --- | --- | --- | --- | --- | --- |
|  |  |  |  | **x** | **y** | **z** |  |  |  |
| Reward probability | 1 | 269 | 6.0478 | -6 | 38 | -8 | Medial frontal gyrus, orbital part | R | 65 |
|  |  |  |  |  |  |  | Medial frontal gyrus, orbital part | L | 54 |
|  |  |  |  |  |  |  | Anterior cingulate cortex | L | 28 |
|  | 2 | 268 | 7.7908 | 33 | 35 | -14 | Inferior frontal gyrus, orbital part | R | 170 |
|  |  |  |  |  |  |  | Inferior frontal gyrus, triangular part | R | 66 |
|  |  |  |  |  |  |  | Middle frontal gyrus | R | 15 |
|  | 3 | 287 | 6.5859 | -39 | 35 | -17 | Inferior frontal gyrus, orbital part | L | 142 |
|  |  |  |  |  |  |  | Inferior frontal gyrus, triangular part | L | 86 |
|  |  |  |  |  |  |  | Middle frontal gyrus, orbital part | L | 11 |
|  | 4 | 901 | 6.4539 | 9 | 62 | 28 | Medial superior frontal gyrus | L | 257 |
|  |  |  |  |  |  |  | Medial superior frontal gyrus | R | 244 |
|  |  |  |  |  |  |  | Superior frontal gyrus | R | 96 |
|  | 5 | 357 | 6.918 | 60 | -55 | 13 | Middle temporal gyrus | R | 133 |
|  |  |  |  |  |  |  | Angular gyrus | R | 45 |
|  |  |  |  |  |  |  | Superior temporal gyrus | R | 42 |
|  | 6 | 663 | 7.2654 | -63 | -58 | 19 | Angular gyrus | L | 192 |
|  |  |  |  |  |  |  | Middle temporal gyrus | L | 70 |
|  |  |  |  |  |  |  | Supramarginal gyrus | L | 68 |
|  | 7 | 15 | 4.1344 | -60 | 23 | 16 | Inferior frontal gyrus, triangular part | L | 13 |
|  | 8 | 204 | 5.9394 | 6 | -25 | 46 | Middle cingulate cortex | L | 89 |
|  |  |  |  |  |  |  | Middle cingulate cortex | R | 63 |
|  |  |  |  |  |  |  | Posterior cingulate cortex | L | 36 |
|  | 9 | 138 | 5.5514 | 45 | -19 | 58 | Precentral gyrus | R | 109 |
|  |  |  |  |  |  |  | Postcentral gyrus | R | 25 |
|  | 10 | 111 | 5.0773 | 15 | -46 | 79 | Postcentral gyrus | R | 71 |
|  |  |  |  |  |  |  | Superior parietal gyrus | R | 24 |
| Reward reception | 1 | 1322 | 7.0673 | -3 | 35 | -14 | Superior frontal gyrus | L | 211 |
|  |  |  |  |  |  |  | Medial frontal gyrus, orbital part | L | 178 |
|  |  |  |  |  |  |  | Medial superior frontal gyrus | L | 163 |
|  | 2 | 53 | 5.0369 | -36 | 35 | -14 | Inferior frontal gyrus, orbital part | L | 37 |
|  |  |  |  |  |  |  | Middle frontal gyrus, orbital part | L | 14 |
|  | 3 | 430 | 6.3312 | 0 | -43 | 34 | Middle cingulate cortex | R | 100 |
|  |  |  |  |  |  |  | Middle cingulate cortex | L | 97 |
|  |  |  |  |  |  |  | Precuneus | L | 80 |
|  | 4 | 31 | 4.4488 | -48 | -76 | 34 | Angular gyrus | L | 13 |
|  | 5 | 21 | 4.5655 | 15 | 47 | 49 | Superior frontal gyrus | R | 13 |
| Reward prediction error | 1 | 27 | 4.4825 | 33 | 20 | -11 | Inferior frontal gyrus, orbital part | R | 15 |
|  |  |  |  |  |  |  | Insula | R | 12 |
|  | 2 | 79 | 5.8288 | -12 | 8 | -11 | Ventral striatum | L | 27 |
|  |  |  |  |  |  |  | Ventral striatum | L | 15 |
|  | 3 | 98 | 5.7815 | 12 | 8 | -8 | Ventral striatum | R | 35 |
|  |  |  |  |  |  |  | Ventral striatum | R | 21 |
|  | 4 | 29 | 4.4477 | -42 | 44 | -2 | Inferior frontal gyrus, triangular part | L | 17 |
