# Supplementary material for: Goal or Gold: Overlapping Reward Processes in Soccer Players upon Scoring and Winning Money
Source: PLoS One. 2015 Apr 15;10(4):e0122798. doi: 10.1371/journal.pone.0122798 (PMC4398371; doi:10.1371/journal.pone.0122798)
Supplement: S1 Table — (DOCX) [file pone.0122798.s003.docx]

**Table S1.** Physical, soccer, and video game attributes of 28 soccer players.

| **Attributes** | **n** | **Min.** | **Max.** | **Mean** | **SD** |
| --- | --- | --- | --- | --- | --- |
| Age (years) | 28 | 18 | 31 | 24.57 | 3.21 |
| Weight (kg) | 28 | 65 | 91 | 77.25 | 7.33 |
| Height (cm) | 28 | 172 | 194 | 181.82 | 6.19 |
| Soccer experience (years) | 28 | 3 | 25 | 17.43 | 4.91 |
| Training/week (h) | 28 | 4 | 12 | 6.57 | 2.13 |
| League* | 28 | 5 | 11 | 8.46 | 1.64 |
| Video game experience (years) | 20 | 4 | 19 | 10.70 | 4.47 |
| Video gaming/week (h) | 20 | 0 | 15 | 2.55 | 3.24 |

n = number of soccer players who answered the question

* = out of eleven possible leagues, where the 1^st^ German league referred to the “1. Bundesliga”
(1^st^ German National league) and the 11^th^ German league referred to the “Kreisliga D” (District league D).
