# Supplementary material for: Goal or Gold: Overlapping Reward Processes in Soccer Players upon Scoring and Winning Money
Source: PLoS One. 2015 Apr 15;10(4):e0122798. doi: 10.1371/journal.pone.0122798 (PMC4398371; doi:10.1371/journal.pone.0122798)
Supplement: S2 Table — (DOCX) [file pone.0122798.s004.docx]

**Table S2.** HEXACO PI-R 200 domain and facet-level scale values of 28 soccer players.

| **Domain-level scale** | **Facet-level scale** | **Min.** | **Max.** | **Mean** | **SD** |
| --- | --- | --- | --- | --- | --- |
| Honesty-Humility | All of the four below combined | 2.31 | 4.28 | 3.43 | 0.51 |
|  | Sincerity | 2.00 | 4.88 | 3.61 | 0.62 |
|  | Fairness | 2.25 | 4.50 | 3.39 | 0.77 |
|  | Greed avoidance | 1.50 | 4.50 | 3.26 | 0.71 |
|  | Modesty | 2.50 | 4.38 | 3.46 | 0.51 |
| Emotionality | All of the four below combined | 2.28 | 4.09 | 2.98 | 0.43 |
|  | Fearfulness | 1.38 | 4.00 | 2.33 | 0.64 |
|  | Anxiety | 1.63 | 4.13 | 3.18 | 0.68 |
|  | Dependence | 1.25 | 4.25 | 2.98 | 0.72 |
|  | Sentimentality | 2.25 | 4.25 | 3.44 | 0.47 |
| Extraversion | All of the four below combined | 1.78 | 4.78 | 3.71 | 0.66 |
|  | Social self esteem | 2.38 | 5.00 | 4.07 | 0.66 |
|  | Social boldness | 1.38 | 4.50 | 3.43 | 0.90 |
|  | Sociability | 1.88 | 4.88 | 3.70 | 0.71 |
|  | Liveliness | 1.50 | 5.00 | 3.74 | 0.81 |
| Agreeableness | All of the four below combined | 2.06 | 3.69 | 2.96 | 0.44 |
|  | Forgiveness | 1.38 | 3.75 | 2.57 | 0.66 |
|  | Gentleness | 1.63 | 4.38 | 3.04 | 0.70 |
|  | Flexibility | 2.13 | 4.13 | 3.00 | 0.49 |
|  | Patience | 1.88 | 4.50 | 3.24 | 0.65 |
| Conscientiousness | All of the four below combined | 2.41 | 3.94 | 3.30 | 0.50 |
|  | Organization | 1.38 | 4.75 | 3.13 | 0.77 |
|  | Diligence | 2.25 | 4.50 | 3.32 | 0.62 |
|  | Perfectionism | 2.38 | 4.38 | 3.47 | 0.53 |
|  | Prudence | 2.25 | 4.50 | 3.22 | 0.67 |
| Openness to Experience | All of the four below combined | 1.94 | 4.16 | 3.14 | 0.49 |
|  | Aesthetic appreciation | 1.88 | 4.13 | 2.76 | 0.65 |
|  | Inquisitiveness | 2.00 | 4.88 | 3.44 | 0.74 |
|  | Creativity | 1.75 | 4.25 | 3.11 | 0.64 |
|  | Unconventionality | 2.13 | 4.75 | 3.24 | 0.58 |
| *Not applicable* | Altruism | 2.63 | 4.50 | 3.79 | 0.48 |

Scoring range: one to five
