# Supplementary material for: Goal or Gold: Overlapping Reward Processes in Soccer Players upon Scoring and Winning Money
Source: PLoS One. 2015 Apr 15;10(4):e0122798. doi: 10.1371/journal.pone.0122798 (PMC4398371; doi:10.1371/journal.pone.0122798)
Supplement: S3 Table — (DOCX) [file pone.0122798.s005.docx]

**Table S3.** Behavioral data from the soccer paradigm, displaying number of shots and passes in the given 80 unclear and 40 clear situations.

| **Situation** | **Decision** | **Min.** | **Max.** | **Mean** | **SD** |
| --- | --- | --- | --- | --- | --- |
| Unclear | Pass | 12 | 78 | 43.00 | 15.32 |
|  | Shoot | 2 | 68 | 36.82 | 15.23 |
| Clear | Pass | 11 | 21 | 19.46 | 1.93 |
|  | Shoot | 19 | 29 | 20.54 | 1.93 |
