# Supplementary material for: Goal or Gold: Overlapping Reward Processes in Soccer Players upon Scoring and Winning Money
Source: PLoS One. 2015 Apr 15;10(4):e0122798. doi: 10.1371/journal.pone.0122798 (PMC4398371; doi:10.1371/journal.pone.0122798)
Supplement: S4 Table — (DOCX) [file pone.0122798.s006.docx]

**Table S4.** Brain activity related to soccer reward probability, reward reception, and reward prediction error (k >10. df = 27).

| **Contrast** | **Region** | **Laterality** | **MNI coordinates** | | | **Cluster size** | **T** | **p(FWE-corr.)** |
| --- | --- | --- | --- | --- | --- | --- | --- | --- |
|  |  |  | **x** | **y** | **z** |  |  |  |
| Reward probability | TPJ | L | -51 | -70 | 34 | 266 | 6.26 | <0.001 |
|  | dlPFC | L | -15 | 50 | 37 | 77 | 5.53 | 0.008 |
|  | TPJ | R | 60 | -52 | 43 | 108 | 5.36 | 0.001 |
|  | MTG | L | -66 | -37 | 1 | 51 | 5.31 | 0.045 |
|  | POG | R | 45 | -25 | 64 | 132 | 5.01 | <0.001 |
|  | TPJ | L | -60 | -49 | 43 | 70 | 4.70 | 0.013 |
| Reward reception | TPJ | L | -39 | -70 | 43 | 141 | 7.04 | <0.001 |
|  | POG | R | 18 | -34 | 67 | 830 | 6.62 | <0.001 |
|  | vmPFC | L | -3 | 47 | -8 | 332 | 6.57 | <0.001 |
|  | Insula | R | 33 | -16 | 4 | 65 | 6.46 | 0.017 |
|  | VS | R | 24 | 5 | -11 | 168 | 6.13 | <0.001 |
|  | VS | L | -12 | 11 | -5 | 180 | 5.69 | <0.001 |
|  | STG | R | 60 | -16 | 10 | 354 | 5.41 | <0.001 |
|  | dlPFC | L | -18 | 32 | 52 | 94 | 5.09 | 0.003 |
|  | POG | L | -66 | -4 | 10 | 175 | 5.04 | <0.001 |
|  | vlPFC | L | -36 | 41 | -8 | 59 | 4.81 | 0.026 |
|  | PCG | L | -24 | -28 | 55 | 131 | 4.71 | <0.001 |
| Reward prediction error | VS | R | 21 | 2 | -11 | 146 | 7.20 | <0.001 |
|  | vmPFC | L/R | 0 | 41 | -8 | 120 | 5.43 | 0.001 |
|  | VS | L | -18 | 2 | -11 | 165 | 5.33 | <0.001 |
|  | TPJ | L | -42 | -70 | 43 | 67 | 5.09 | 0.017 |
|  | STG | R | 60 | -19 | 7 | 142 | 5.02 | <0.001 |

Abbreviations: dlPFC (dorsolateral prefrontal cortex), MTG (middle temporal gyrus), PCG (precentral gyrus), POG (postcentral gyrus), STG (superior temporal gyrus), TPJ (temporal parietal junction), vlPFC (ventrolateral prefrontal cortex), vmPFC (ventromedial prefrontal cortex), VS (ventral striatum).
