# Supplementary material for: Goal or Gold: Overlapping Reward Processes in Soccer Players upon Scoring and Winning Money
Source: PLoS One. 2015 Apr 15;10(4):e0122798. doi: 10.1371/journal.pone.0122798 (PMC4398371; doi:10.1371/journal.pone.0122798)
Supplement: S5 Table — (DOCX) [file pone.0122798.s007.docx]

**Table S5.** Brain activity related to monetary reward probability, reward reception, and reward prediction error (k >10, df = 27).

| **Contrast** | **Region** | **Laterality** | **MNI coordinates** | | | **Cluster size** | **T** | **p(FWE-corr.)** |
| --- | --- | --- | --- | --- | --- | --- | --- | --- |
|  |  |  | **x** | **y** | **z** |  |  |  |
| Reward probability | vlPFC | R | 33 | 35 | -14 | 268 | 7.79 | <0.001 |
|  | TPJ | L | -63 | -58 | 19 | 663 | 7.27 | <0.001 |
|  | MTG | R | 60 | -55 | 13 | 357 | 6.92 | <0.001 |
|  | vlPFC | L | -39 | 35 | -17 | 287 | 6.59 | <0.001 |
|  | dlPFC | R | 9 | 62 | 28 | 901 | 6.45 | <0.001 |
|  | vmPFC | L | -6 | 38 | -8 | 269 | 6.05 | <0.001 |
|  | MCC | R | 6 | -25 | 46 | 204 | 5.94 | <0.001 |
|  | POG | R | 45 | -19 | 58 | 138 | 5.55 | <0.001 |
|  | POG | R | 15 | -46 | 79 | 111 | 5.08 | 0.001 |
| Reward reception | vmPFC | L | -3 | 35 | -14 | 1322 | 7.07 | <0.001 |
|  | PCC | L/R | 0 | -43 | 34 | 430 | 6.33 | <0.001 |
| Reward prediction error | VS | L | -12 | 8 | -11 | 79 | 5.83 | 0.011 |
|  | VS | R | 12 | 8 | -8 | 98 | 5.78 | 0.004 |

Abbreviations: dmPFC (dorsomedial prefrontal cortex), MCC (Midcingulate cortex), MTG (middle temporal gyrus), POG (postcentral gyrus), TPJ (temporal parietal junction), vlPFC (ventrolateral prefrontal cortex), vmPFC (ventromedial prefrontal cortex), VS (ventral striatum).
