# Supplementary material for: Goal or Gold: Overlapping Reward Processes in Soccer Players upon Scoring and Winning Money
Source: PLoS One. 2015 Apr 15;10(4):e0122798. doi: 10.1371/journal.pone.0122798 (PMC4398371; doi:10.1371/journal.pone.0122798)
Supplement: S6 Table — (DOCX) [file pone.0122798.s008.docx]

**Table S6.** Differential Brain activity upon contrasting soccer versus monetary reward reception
(paired t-test, k >10, df = 27).

| **Contrast** | **Region** | **Laterality** | **MNI coordinates** | | | **Cluster size** | **T** | **p(FWE-corr.)** |
| --- | --- | --- | --- | --- | --- | --- | --- | --- |
|  |  |  | **x** | **y** | **z** |  |  |  |
| Soccer versus monetary reward reception | TPJ | R | 36 | -37 | 43 | 674 | 7.48 | <0.001 |
|  | vlPFC | L | -39 | 62 | -5 | 71 | 5.20 | 0.011 |
|  | TPJ | L | -36 | -55 | 52 | 126 | 4.92 | <0.001 |
|  | TPJ | L | -24 | -61 | 43 | 52 | 4.87 | 0.038 |

Abbreviations: TPJ (temporal parietal junction), vlPFC (ventrolateral prefrontal cortex)
