# Supplementary material for: Goal or Gold: Overlapping Reward Processes in Soccer Players upon Scoring and Winning Money
Source: PLoS One. 2015 Apr 15;10(4):e0122798. doi: 10.1371/journal.pone.0122798 (PMC4398371; doi:10.1371/journal.pone.0122798)
Supplement: S7 Table — (DOCX) [file pone.0122798.s009.docx]

**Table S7.** Negative correlation between the HEXACO PI-R 200 Honesty-Humility scale and brain activity at scoring after a pass versus scoring after a shot (k >10, df = 27).

| **Contrast** | **Region** | **Laterality** | **MNI coordinates** | | | **Cluster size** | **T** | **p(FWE-corr.)** |
| --- | --- | --- | --- | --- | --- | --- | --- | --- |
|  |  |  | **x** | **y** | **z** |  |  |  |
| Goal after a pass versus goal after a shot | Middle frontal gyrus | L | -27 | 8 | 40 | 104 | 4.93 | 0.002 |
