# Supplementary material for: Goal or Gold: Overlapping Reward Processes in Soccer Players upon Scoring and Winning Money
Source: PLoS One. 2015 Apr 15;10(4):e0122798. doi: 10.1371/journal.pone.0122798 (PMC4398371; doi:10.1371/journal.pone.0122798)
Supplement: S8 Table — (DOCX) [file pone.0122798.s010.docx]

**Table S8.** Brain region name abbreviations.

| **Region name abbreviation** | **Full region name** |
| --- | --- |
| dlPFC | Dorsolateral prefrontal cortex |
| dmPFC | Dorsomedial prefrontal cortex |
| Ins | Insula |
| MCC | Midcingulate cortex |
| MFG | Middle frontal gyrus |
| mOFC | Orbitofrontal cortex |
| MTG | Middle temporal gyrus |
| mOFC | Orbitofrontal cortex |
| PCC | Posterior cingulate cortex |
| PCG | Precentral gyrus |
| POG | Postcentral gyrus |
| rACC | Rostral anterior cingulate cortex |
| sgACC | Subguneal anterior cingulate cortex |
| STG | Superior temporal gyrus |
| TPJ | Temporal parietal junction |
| vlPFC | Ventrolateral prefrontal cortex |
| vmPFC | Ventromedial prefrontal cortex |
| VS | Ventral striatum |

Neuroscientific brain region descriptions:

Regions often used in the literature were summarized as functional entities. Of these, activations in the VS were summarized as activations of the ventral anterior regions of the putamen and the caudate nucleus. Additionally, the TPJ was considered to include the supramarginal and angular gyrus, as well as the inferior parietal lobule. Activations of the superior frontal gyrus, the medial part of the superior frontal gyrus, the orbital part of the superior frontal gyrus, the orbital part of the medial frontal gyrus, and the orbital part of the superior frontal gyrus were referred to as activations of the dlPFC, dmPFC, mOFC, vlPFC, or vmPFC. The regional definitions were performed only upon closer inspection of each regional activation.
